# Supplementary material for: A Study of the Mechanisms and Characteristics of Fluorescence Enhancement for the Detection of Formononetin and Ononin
Source: Molecules. 2023 Feb 5;28(4):1543. doi: 10.3390/molecules28041543 (PMC9958771; doi:10.3390/molecules28041543)
Supplement: Supplementary file 1 [file molecules-28-01543-s001.zip › molecules-2157695-supplementary.pdf]

Supporting Information

Studies of Mechanisms and Characteristics of the Fluorescence Enhancement for the Detection of Formononetin and Ononin

Jinjin Cao[1,†], Tingting Li[1,†], Ting Liu[1], Yanhui Zheng [3*], Jiamiao Liu[1], Qifan Yang [1], Xuguang Li[1], Wenbo Lu [2*], Yongju Wei [3], Wenhong Li,[1*]

[1] Department of Environmental and Chemical Engineering, Hebei College of Industry and Technology, Shijiazhuang 050091, China

2 Key Laboratory of Magnetic Molecules and Magnetic Information Materials (Ministry of Education), School of Chemistry and Material Science, Shanxi Normal University, Taiyuan 030031, China

3 College of Chemistry and Material Science，Hebei Normal University，Shijiazhuang 050024, China

* Correspondence: luwb@sxnu.edu.cn (W. L.); zhengyh0308@163.com(Y. Z.); liwenhong2006@126.com (W. L.);Tel.: (86)13784038302

Contents

Figure S1. ¹H NMR of Compound C … 1
Figure S2. HRMS of Compound C[M+H] …. 2
Figure S3. ¹H NMR of Compound B’..2
Figure S4. HRMS of Compound B’[M+NH4] 3

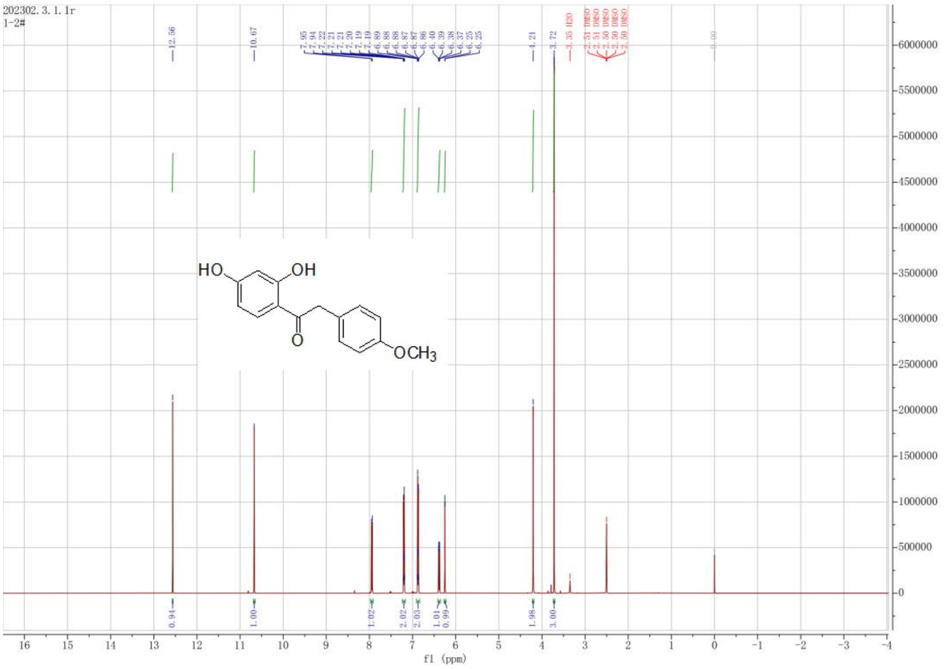

Figure S1. ¹H NMR of Compound C

¹H NMR analysis were performed using WNMR-I 400MHz nuclear magnetic resonance spectrometer of Wuhan Zhongke Oxford Spectroscopy Technology Co., Ltd.

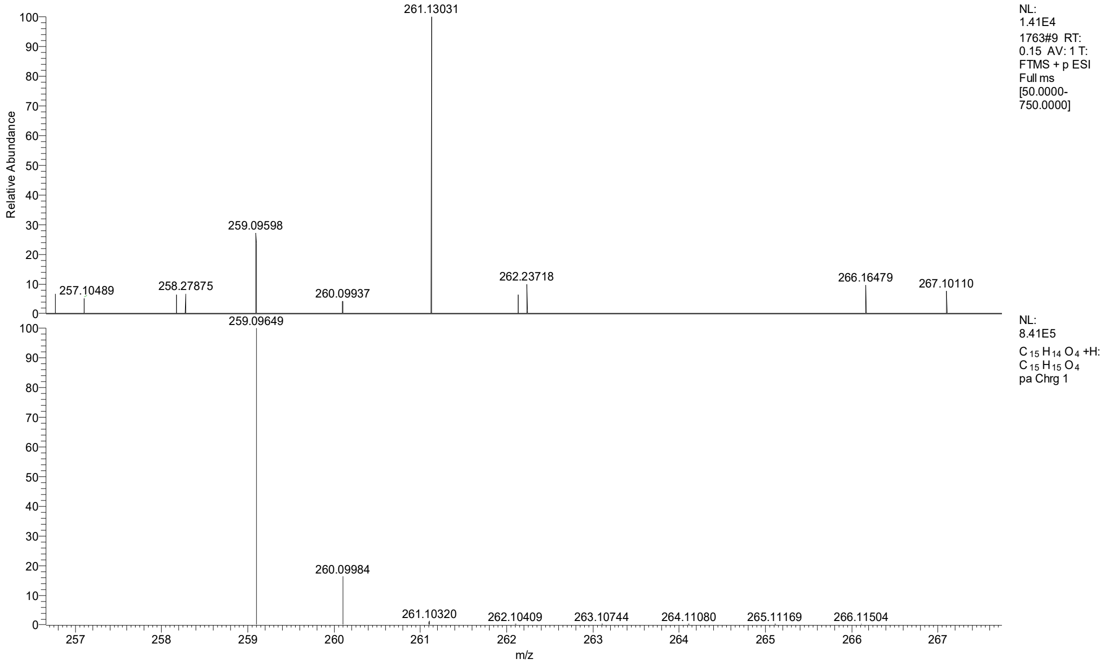

Figure S2. HRMS of Compound C[M+H]

HRMS analysis were performed using Thermo Scientific Q Exactive Orbitrap.

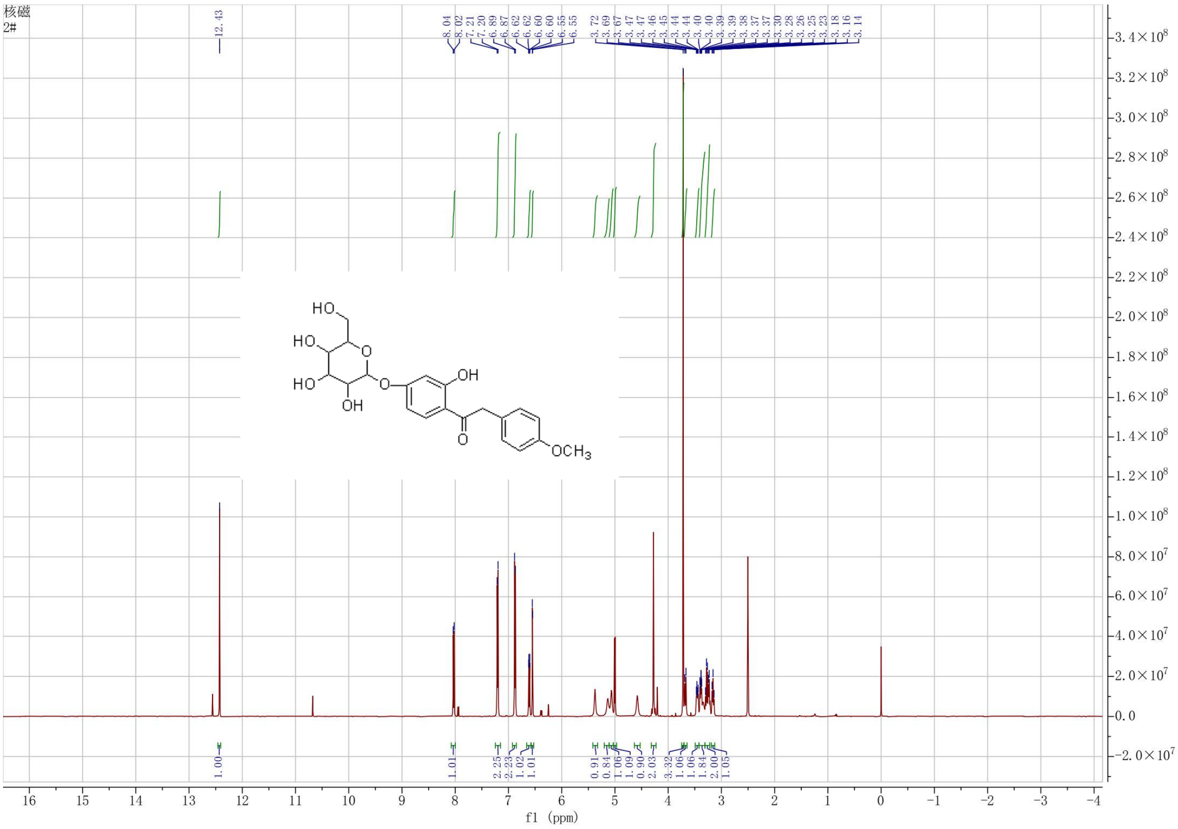

Figure S3. ^{1}H NMR of Compound B′

^{1}H NMR analysis were performed using WNMR-I 400MHz nuclear magnetic resonance spectrometer of Wuhan Zhongke Oxford Spectroscopy Technology Co., Ltd.

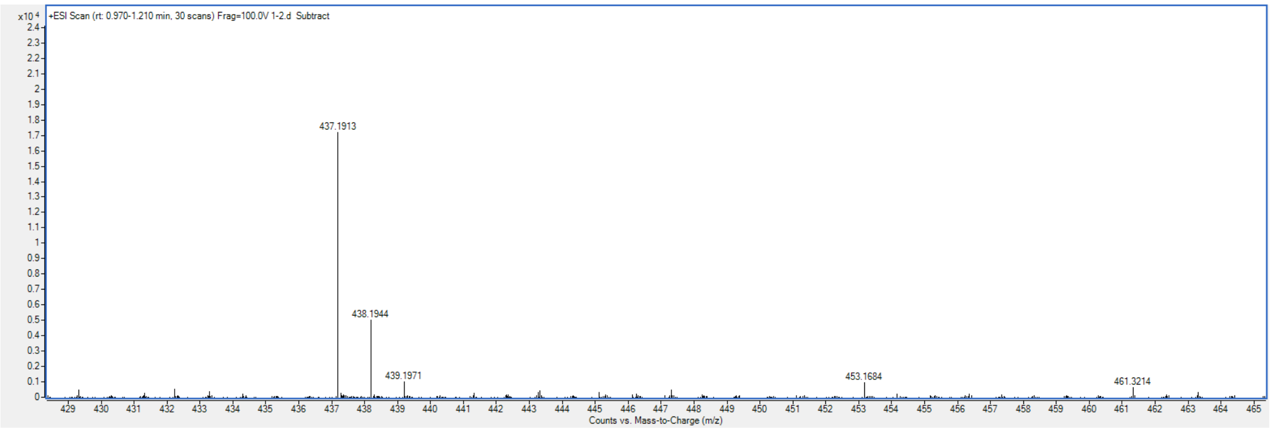

Figure S4. HRMS of Compound B'[M+NH4]

HRMS analysis were performed using Agilent 1290II/6545 Liquid Chromatography Mass Spectrometer.
